# Kidney Diseases

**Published:** 1913-03

**Authors:** 


**Kidney Diseases.** By W. P. HERRINGHAM, M.D., F.R.C.P. Pp. xvi, 378. London: Oxford Medical Publications. 1912. Price, 15s. net.

The author of this book has given us a most interesting volume based on his own observations, the result of many years of work. All aspects of renal disease are very fully dealt with, though unfortunately little that is fresh is added in the way of treatment. Nevertheless he considers that there is no disease in which more can be done for the good of the patient by careful treatment than can be done in nephritis. The author's views on the relation of the different forms of nephritis to each other and to other morbid states are full of interest, and very clearly expounded. He explains his meaning by typical clinical cases, the pathology and morbid anatomy of which are admirably illustrated. We should, however, have liked to have heard something of the condition of the suprarenal glands in these cases.

After discussing the cause of uræmia at some length, the author suggests that the phenomena may possibly be connected with the absorption of dying cells, with the production of a condition analagous to anaphylaxis. His next paragraph, however, appears to subvert this, for it states that uræmic symptoms rarely occur in the large white fatty kidney, but are chiefly seen in those forms of the disease which are accompanied by fibrosis. This does not agree with our experience, for out of a consecutive series of 215 deaths from chronic renal disease 66 per cent. of the patients who suffered from the parenchymatous form of the disease died from uræmia, while only 13 per cent. of those who had typical granular kidneys died from the same cause.

With reference to tuberculin treatment in renal tuberculosis, the author appears to have little faith, and says that the more familiar vaccine therapy becomes the more hesitatingly are its claims put forward.

Several chapters devoted to renal disorders in connection with pregnancy have been written by Herbert Williamson, who has performed his work well, explaining most lucidly the modern views on the origin, course and treatment of these toxæmias.

